# Host range of 30 novel *Arthrobacter globiformis* bacteriophages shows modularity and nestedness

**DOI:** 10.17912/micropub.biology.002306

**Published:** 2026-08-22

**Authors:** Yu-Chuan Chen, Emily Chung, Kristen DeJanes, Kiri Diaz-Asper, Anna Hayashizaki, Valerie Jackson, Nikhita Lalwani, John Lin, Martin Lin, Sarah Liu, Taylor Palmore, Rouida Siddiqui, Neron Xavier, Nic M. Vega

**Affiliations:** 1 Department of Biology, Emory University, Atlanta, GA, United States; 2 Department of Physics, Emory University, Atlanta, GA, United States

## Abstract

The SEA-PHAGES program maintains an extensive archive of student-discovered Actinobacteriophage as a resource for understanding phage diversity. Here, we examine host range of thirty phages isolated on *Arthrobacter globiformis* B-2979 against additional strains of *A. globiformis* and against other isolates within ‘ *Arthrobacter sensu stricto* '. Consistent with other reports, host range of these phages was generally narrow. A general relationship between magnitude and prevalence was observed in efficiency of plating on *globiformis* isolates other than the isolation host, suggesting underlying mechanisms of cross-infectivity.

**Figure 1. Host range of 30 phages isolated on A. globiformis B-2979:** (A) Frequency of plaque formation on test *Arthrobacter* isolates. Genus of the test host is shown at the top of each panel. Height of each bar indicates the fraction of phage isolates forming any plaques (including LFW) on each host. No plaques or lysis from without (LFW) events were observed on *A. pascens* (B-1814, B-2884); one phage (IllegallySmol) showed LFW but not countable plaques on *A. humicola* B-24479. (B) Efficiency of plating (EOP) on test bacteria. Instances of lysis from without (LFW, open triangles) are shown by allowing a count of one plaque for the least dilute spot where any plaquing was observed; these values do not represent exact counts, are excluded from statistical tests, and are shown for comparison only. A sub-set of combinations from the initial assay (run 1, left) were re-tested in an independent experiment (run 2, right) to show repeatability. Host range assessment was repeatable, with higher run to run variation at lower EOPs. (C) Heatmap of log _10_ (EOP) by phage isolate x host, in run 1 (left) and run 2 (right). Combinations that did not produce detectable plaque formation are shown in grey. Combinations not performed are shown in white. (D) log _10_ (EOP) relationships among non-host bacteria. Each data point represents one phage isolate; combinations of phage and host for which plaques were not observed were given a value of -8 to allow visualization. Black horizontal and vertical lines indicate EOP=1; grey line is 1:1; red dashed lines represent linear fits to log _10_ (EOP) data from phages with measurable and reliable EOP (≥10 ^-4^ ) on both hosts. Only the relationship between *globiformis* strains B-2880 and B-24025 had statistical support (log _10_ (B-2880 EOP) = 0.7004 * log _10_ (B-24025 EOP) – 0.9371, adjusted R ^2^ = 0.76, F test p=1.43*10 ^-4^ , df=10). All phage stocks had estimated titers ≥10 ^9^ PFU/mL on isolation host B-2979, except Coopy at 8e ^8^ and IllegallySmol at 9e ^8^ . Two phage stocks isolated from the same sample were subsequently identified as being the same phage (Atlantica) based on detection PCR, and these data sets were combined.

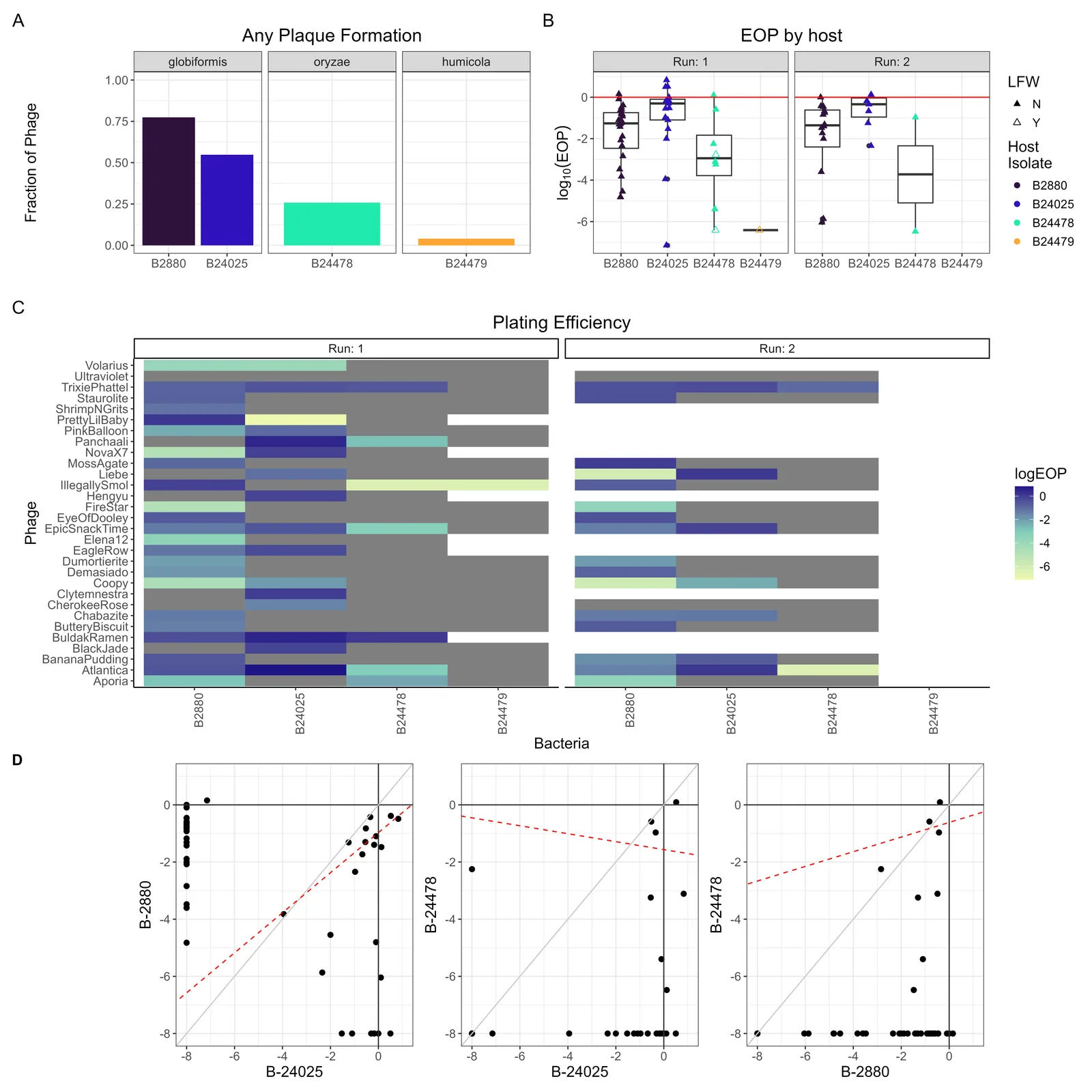



## Description

Host range is an important aspect of phage ecology. Many isolated phages infect few hosts outside the type used for isolation, at least in part due to over-representation of narrow host range phages by conventional isolation methods (Jensen et al., 1998). Even so, phage-host specificity varies across host clades and across phages isolated on a given host (Gencay et al., 2019; Xie et al., 2018), and very broad host ranges are possible (Malki et al., 2015). “Modularity” is common, where sets of phage isolates infect shared sets of hosts, with few productive interactions outside that range (Beckett & Williams, 2013; Flores et al., 2013; Göller et al., 2021; Holtappels et al., 2023), as is nestedness, where progressively more specialized phage infect progressively less permissive hosts (Flores et al., 2011).

The SEA-PHAGES program (Jordan et al., 2014) has generated new insights into phage diversity. Hosts from the genus *Arthrobacter* have been added to the program in recent years; most of the phages infecting this genus were isolated on one of four isolates of *Arthrobacter globiformis* (1711 of 2640 *Arthrobacter* phages archived; 353 of 635 sequenced). Host range of *Arthrobacter* phages is generally narrow, often limited to the isolating host and sometimes extending to a small number of closely related strains (Brown et al., 1978; Einck et al., 1973; Germida & Casida, 1981). Recent work has supported this idea, with phage isolates plaquing on 0-18% of *Arthrobacter* species apart from the isolation host (Kaliniene et al., 2017; Klyczek et al., 2017). However, prior studies have generally focused on cross-infectivity across bacterial species; very few productive infections are observed at this range, and relationships in cross-infectivity are difficult to determine.

In these experiments, we determined host range of thirty phages isolated on *A. globiformis* B-2979 against a set of six non-isolation host isolates (“test bacteria”) within ‘ *Arthrobacter sensu stricto'* (Busse, 2016) (Table 1), including two additional strains of *A. globiformis* (B-2880, B-24025). Twenty-seven phages were isolated in or near Atlanta, Georgia; three external phages (Liebe, Ultraviolet, and TrixiePhattel) were not exceptions to the patterns observed.

Phages created countable plaques on zero (phage Ultraviolet only), one (n=19), two (n=6), or three (n=5) test bacteria. No plaque formation was observed for any of these phages on either isolate of *A. pascens.* Most successful infections were against strains of *A. globiformis* ( Fig. 1A ). While cross-infection on *A. globiformis* B-2880 was more common than on B-24025 ( Fig. 1A ), efficiency of plating (EOP) tended to be higher on B-24025 than on B-2880 (median log _10_ EOP for B-24025 = -0.32; median log _10_ EOP for B-2880 = -1.31) ( Fig. 1B ). A second, independent run of the host range assay using a sub-set of phages and hosts indicated that host range data were largely replicable, with most of the variation in phage-host combinations which produced a low EOP (~10 ^-4^ or lower) ( Fig. 1B- C).

EOP was highly variable both within and among phages. Among the minority of phages that infected both test *globiformis* , the same pattern held as in (1B), with EOP on B-2880 being in general lower than EOP on B-24025 for the same phage ( Fig. 1D ). However, three phages showed markedly higher EOP on B-2880 than on B-24025, showing that this general trend need not hold for specific cases (Coopy, two replicates, log _10_ EOP B-24025 ≈ -2 and B-2880 ≈ -4.5; Liebe, two replicates, log _10_ EOP B-4025 ≈ -1 and B-2880 ≈ -6; NovaX7, one replicate, log _10_ EOP B-4025 = -0.1 and B-2880 =-4.8). Likewise, one phage is substantially off the line in the opposite direction (PrettyLilBaby, one replicate, log _10_ EOP B-2880 = 0.15 and B-24025 = -7.1). Phages successfully infecting *A. oryzae* B-24478 also infected at least one and often both test *globiformis* ( Fig. 1D ) *,* suggesting generalist tendencies in a subset of phage isolates.

These results are consistent with previous observations of narrow host range in *Arthrobacter* phages and are suggestive of modularity and/or nestedness; larger data sets containing phages from multiple target hosts are required to test these ideas. Further, the observed relationship between magnitude and prevalence of EOP on two test *globiformis* suggests underlying mechanism(s) of cross-susceptibility, with notable departures from the rule that may prove informative. As the current data set is small, and as few of these phages (and none of these hosts) are currently sequenced, it is difficult to hypothesize specific mechanisms. However, the available data indicate that distantly related phages can have similar host ranges ( Fig. 1C ). For example, the sequenced siphoviral phages Atlantica (temperate, cluster AS3) (Bakayoko et al., 2026; Jackson & Vega, 2025) and TrixiePhattel (virulent, cluster AU6) (Hernandez et al., 2026; Wise & Sivanathan, 2025) show relatively broad host ranges, with plaque formation on both test *globiformis* as well as on *A. oryzae* . It is reasonable to hypothesize that some features of relatedness will explain some of the variation in host range, but the relationship(s) between genetic distance, genome content, and host range in these phages remains an open question.

## Methods

*Media and buffers.* LB broth (per L: 10 g peptone, 5 g yeast extract, 5 g NaCl) was used for growth of bacteria. Modified PYCa growth medium (per L: 1g yeast extract, 15 g peptone, 4.5 mM CaCl _2_ , 10 mM MgSO _4_ , 0.1% dextrose) was used as broth, base agar (1.5%) and top agar (0.3%), with cycloheximide (10 µg/mL) to inhibit fungal growth. Phage buffer (10 mM Tris pH 7.5, 10 mM MgSO _4_ , 68 mM NaCl, 1 mM CaCl _2_ , 10% glycerol) was used for suspensions and serial dilutions (Zorawik et al., 2024).

*Bacterial culture* . Bacterial cultures were created by scraping single colonies from plates to inoculate 2 mL LB growth medium, which was grown for 48h at 25°C with shaking at 200 RPM. Stationary phase cultures were diluted 1:1000 into PYCa and grown overnight under the same conditions to acclimate to the medium, then re-inoculated 1:25 into PYCa and returned to incubation for 4-6h to create log-phase cultures.

*Phage isolation.* Phages were isolated as part of the Science Education Alliance-Phage Hunters Advancing Genomics and Evolutionary Science (SEA-PHAGES) program (Jordan et al., 2014) using common procedures (Zorawik et al., 2024). All phages were isolated with target host *Arthrobacter globiformis* B-2979 and plating medium PYCa. Phages isolated at Emory used an incubation temperature of 30°C in 2023-2024 and 22°C in 2025. Briefly, 15 mL PYCa was added to 15 mL of soil in a 50 mL conical tube, then incubated with shaking at 200 RPM for ~4 hours. Soil particles and debris were removed through centrifugation (2000xg for 10 min) and filtration of supernatant (0.22 µm pore size). Filtrate was mixed 1:1 with top agar + 100 µL B-2979 for direct plating, and the remaining volume was enriched with 1:500 v/v B-2979 and allowed to incubate for 48h with shaking. The enriched culture was centrifuged to pellet bacteria, and the filtered supernatant (0.22 µm) was used for spot and/or full double layer plates with top agar enriched with 20-100 µL of B-2979. Phages were purified through 2-4 rounds of plaque picking and re-plating to obtain homogeneous stocks.

*Host range.* Host range assays were carried out using common procedures (Zorawik et al., 2024). Briefly, double layer plates were created with 50-100 µL of each log-phase bacterial culture, 1 mL of phage buffer (10% glycerol), and 3 mL of top agar and allowed to completely solidify before plating 3 µL spots of 10-fold serially diluted phage. Plates were incubated at 25-30°C for 24-48h before counting. Efficiency of plating (EOP) was calculated as [PFU on test bacteria]/[PFU on B-2979]. Lysis from without (LFW) was recorded when confluent spots but not single plaques were observed.

*Data analysis* . Data from all users and runs was combined for analysis in R (v4.4.0), using data handling functionality from *tidyverse* v2.0.0 ( *stringr* v1.5.1, *dplyr* v1.1.4) (Wickham et al., 2019) and plotting with *ggplot* v3.5.1, *ggpubr* v0.6.0, *cowplot* v1.1.3, and *patchwork* v1.2.0 (Kassambara, 2020; Wickham, 2016; Wilke, 2020). Statistical functions *lm()* and *wilcox.test()* were provided by package *stats* in base R. Data and code are available at https://github.com/veganm/ArthrobacterPhageHostRange

## Reagents

*Bacterial isolates*

**Table:** 

**Strain**	**Genotype**	**Available from**
B-2979	*Arthrobacter globiformis* (target host)	USDA NRRL; SEA-PHAGES
B-1814	*Arthrobacter pascens*	USDA NRRL; SEA-PHAGES
B-2880	*Arthrobacter globiformis*	USDA NRRL; SEA-PHAGES
B-2884	*Arthrobacter pascens*	USDA NRRL
B-24025	*Arthrobacter globiformis*	USDA NRRL; SEA-PHAGES
B-24478	*Arthrobacter oryzae*	USDA NRRL; SEA-PHAGES
B-24479	*Arthrobacter humicola*	USDA NRRL

*Phage isolates*

**Table:** 

**Date**	**Institution**	**PhagesDB Name**	**Cluster**	**Latitude**	**Longitude**
2023	Emory	Coopy	Not Sequenced	33.795761	-84.320096
2023	Emory	BlackJade	FL	33.795761	-84.320096
2023	Emory	MossAgate	Not Sequenced	33.79158	-84.31657
2023	Emory	Staurolite	Not Sequenced	33.7969051	-84.3178155
2023	Emory	ShrimpNGrits	Not Sequenced	33.7957465	-84.3203161
2023	Emory	ButteryBiscuit	Not Sequenced	33.7957465	-84.3203161
2023	Emory	Chabazite	Not Sequenced	33.795831	-84.320273
2023	Emory	Dumortierite	Not Sequenced	33.795937	-84.321014
2023	Emory	Demasiado	Not Sequenced	33.795937	-84.321014
2024	Emory	Volarius	Not Sequenced	33.794812	-84.327572
2024	Emory	PinkBalloon	Not Sequenced	33.791944	-84.328611
2024	Emory	IllegallySmol	Not Sequenced	33.796000	-84.321180
2024	Emory	LoreleiDooley	Not Sequenced	33.796000	-84.321180
2024	Emory	FireStar	Not Sequenced	33.796000	-84.321180
2024	Emory	Clytemnestra	Not Sequenced	33.796000	-84.321180
2024	Emory	Aporia	Not Sequenced	33.796000	-84.321180
2024	Emory	BananaPudding	Not Sequenced	33.796000	-84.321180
2024	Emory	Panchaali	FC	33.796000	-84.321180
2024	Emory	Atlantica	AS3	33.796720	-84.323940
2024	Emory	EpicSnackTime	Not Sequenced	33.796720	-84.323940
2024	Emory	CherokeeRose	Not Sequenced	33.796720	-84.323940
2017	University of Pittsburg	Liebe	AZ2	40.4415	-79.9503
2024	HHMI	TrixiePhattel	AU6	34.432769	-112.415035
2024	NCSU	Ultraviolet	Not Sequenced	39.203739	-76.684837
2025	Emory	BuldakRamen	Not Sequenced	33.47311	- 84.19250
2025	Emory	EagleRow	Not Sequenced	33.79582	-84.32135
2025	Emory	Elena12	Not Sequenced	33.79589	-84.32736
2025	Emory	Hengyu	Not Sequenced	33.79061	-84.32791
2025	Emory	NovaX7	Not Sequenced	33.79095	-84.30056
